# 

**DOI:** 10.1038/sj.bjc.6600397

**Published:** 2002-01-02


					Author Index
Aapro M S4 (S2)
Abdallah SY S45 (P37)
Aberdeen Lung Cancer Group
S61 (P91)
Aboagye EO S13 (1.2), S14
(1.5), S110 (P251)
Abraham J S47 (P46)
Abraham JE S71 (P121)
Adams C S114 (P265)
Adams D S26 (6.3)
Adams EF S17 (2.6)
Adams M S72 (P126)
Adlard JW S77 (P141), (P142)
Agarwal R S44 (P35)
Agarwall R S60 (P86)
Agathanggelou A S92 (P192)
Agrawal R S72 (P126)
Ahamed E S61 (P91)
Aherne GW S79 (P147)
Airley RE S28 (7.2)
Akubani G S89 (P182), S106
(P239)
Al-Assah RS S96 (P205)
Al-Sakkaf KA S114 (P264)
Alcorn MJ S18 (3.4)
Alderson D S7 (S16)
Alexander-Sefre F S42 (P29)
Allan E S27 (6.6)
Allen C S28 (7.1)
Allen O S47 (P47)
Allen W S21 (4.4)
Ameer-Beg SM S81 (P156)
Amess J S40 (P24)
Andrews H S4 (S4), S85
(P169)
Angerson WJ S42 (P28)
Anthoney A S60 (P88)
Appleyard MVCL S39 (P19),
S89 (P183), S113 (P262)
Arcaro A S120 (P283)
Ardern-Jones A S24 (5.4), S70
(P119)
Argraves WS S32 (8.6), S98
(P213)
Ashcroft L S68 (P113)
Ashley S S18 (3.2)
Ashman JNE S59 (P84)
Ashton SE S99 (P216)
Athavale RD S43 (P31)
Atkinson RJ S44 (P34)
Australasian Leukaemia &
Lymphoma Group S1
(CT4)
Australian Bladder Cancer
Study Group S23 (5.1)
Avizienyte E S119 (P279)
Awan A S48 (P50)
Azim-Araghi A S111 (P256)
Bahia H S59 (P84)
Baig S S100 (P218), S102
(P223)
Bailey C S2 (CT7)
Bakkevold KE S1 (CT3)
Baldwin DR S61 (P92)
Bali A S113 (P261)
Balkwill F S38 (P15), S45
(P39)
Ball D S72 (P125)
Ball KL S38 (P16)
Bamberger ES S45 (P38)
Bandara MJ S16 (2.2)
Bannister A S8 (S20)
Barnes DM S70 (P119)
Barnett GC S58 (P81)
Barr MP S98 (P211)
Barry T S22 (4.6)
Barsacq G S103 (P228)
Barth JL S32 (8.6)
Barthel H S13 (1.2)
Bartlett JMS S92 (P190)
Barton DE S91 (P189)
Baselga J S114 (P266)
Bashborun A S18 (3.1)
Bass A S110 (P253)
Basu B S47 (P46)
Bateman AC S32 (8.4)
Bauer U-M S8 (S20)
Bauer W S115 (P268)
Beechey-Newman N S18 (3.1),
S35 (P6), (P7), (P8)
Begent RHJ S100 (P218), S102
(P223), S117 (P272), S112
(P257)
Bell S S1 (CT1), S20 (4.2)
Bellamy EA S34 (P3)
Benghiat A S69 (P117)
Benson J S34 (P4)
Benson RJ S71 (P121)
Beresford M S54 (P67)
Beresford MJ S66 (P107)
Berg K S110 (P252), (P253)
Bergin OE S74 (P132)
Berry JM S117 (P273)
Betts CD S67 (P109)
Beynon J S73 (P129), (P130)
Bhala N S65 (P102)
Bhana R S20 (3.8)
Bibby MC S104 (P230), S106
(P238), S112 (P259), S116
(P270)
Bigley A S100 (P217)
Bilsland A S103 (P227)
Birchall LJ S109 (P250)
Birtle AJ S66 (P107)
Bissett D S61 (P91)
Bissett JD S67 (P110)
Blakey DC S99 (P216)
Blann A S42 (P30)
Blenkiron C S90 (P184)
Boddy AV S118 (P276)
Boden R S112 (P257)
Boesen A-M S1 (CT4)
Bolton S S57 (P76)
Bonnar J S46 (P40)
Boonsong A S32 (8.5)
Borsellino N S61 (P89)
Boshoff C S11 (S28)
Botting KJ S30 (7.8)
Bouchier-Hayes D S21 (4.5),
S79 (P148), S98 (P212),
(P211)
Bouchier-Hayes DJ S98
Boulos N S91 (P188)
Bowen RL S100 (P218), S102
(P223)
Bower M S19 (3.5), S60 (P87),
S63 (P97)
Bowman KJ S33 (8.7), S112
(P260)
Box G S96 (P203)
Boxall J S99 (P215)
Boxall K S79 (P147)
Boxer GM S112 (P257)
Boyd M S6 (S12), S57 (P77),
S59 (P82), (P83), S89
(P182), S106 (P239)
Boyer J S21 (4.4)
Boyne J S101 (P221)
Brader S S96 (P203)
Bradshaw TD S15 (2.1), S108
(P245), S116 (P270), S117
(P273)
Brady ME S85 (P168)
Brave SR S99 (P216), S100
(P217)
Braybrooke J S27 (6.7), S38
(P15), S42 (P30)
Braybrookes J S114 (P266)
Breathnach F S40 (P23)
Briggs C S64 (P101)
Briggs CH S67 (P109)
Briggs T S58 (P79)
Brigham J S59 (P84)
Brimmell M S32 (8.4)
Britton J S33 (8.8)
Britton K S26 (6.2)
Brookes R S115 (P267)
Brooks AC S86 (P173)
Browett P S1 (CT4)
Brown BL S114 (P264)
Brown E S62 (P95)
Brown G S13 (1.2)
Brown J S1 (CT1), S3 (CT8),
S23 (5.2)
Brown JE S19 (3.6)
Brown JM S2 (CT6)
Brown LM S97 (P206)
Brown MM S59 (P82)
Brown R S9 (S23), (S24), S16
(2.2), S20 (4.1), S103
(P226)
Brown SM S26 (6.5)
Browne P S40 (P23), S88
(P180)
Browne S S115 (P267)
Brownlie A S104 (P232)
Brunton VG S119 (P279)
Bryan R S74 (P131)
Buckels J S30 (7.6)
Buggy Y S87 (P175)
Bulusu RV S54 (P69)
Bulusu VR S47 (P46), (P47)
Burchill S S80 (P152), S101
(P221)
Burchill SA S95 (P201), (P202)
Burdall S S22 (4.7)
Burger AM S117 (P273)
Burke F S45 (P39)
Burke MD S117 (P274)
Burnet K S34 (P4)
Burnet NG S54 (P69), S55
(P70)
Burney IA S46 (P41)
Burniston M S68 (P114), S69
(P115)
Burns PA S33 (8.7)
Buscombe JR S100 (P218),
S102 (P223)
Butler D S48 (P48)
Butler PC S117 (P274)
Cade J S51 (P58)
Cairns SC S120 (P284)
Calman F S62 (P95)
Calvete JA S99 (P216)
Cameron D S40 (P22), S50
(P54)
Camplejohn RS S70 (P119)
Cannon S S58 (P79)
Carey F S46 (P42)
Carmichael J S38 (P17), S49
(P53), S108 (P245)
Carney D S91 (P189)
Carragher NO S76 (P140)
Carrington BM S26 (6.2)
Carroll P S88 (P180)
Caruso M S61 (P89)
Cassidy J S32 (8.5), S52 (P61),
S88 (P178), S120 (P285)
Catchatourian R S39 (P20),
(P21)
Cattell E S114 (P265)
Cawkwell L S59 (P84)
Chadderton N S29 (7.4)
Chalmers A S31 (8.3)
Chalmers D S51 (P59)
Chamberlain MP S39 (P19),
S89 (P183)
Chan A S46 (P42)
Chan D S75 (P135), S82 (P159)
Chan FL S92 (P191)
Chan PSF S92 (P191)
Chaplin DJ S118 (P278)
Charlton P S22 (4.8)
Cheah KSE S75 (P135), S82
(P159)
Chen G S92 (P191)
Chen S S11 (S31)
Chenevert TL S5 (S9)
Chester K S117 (P272)
Cheung KMC S75 (P135)
Chew GK S24 (5.6)
Child T S1 (CT1)
Childs K S117 (P275)
Chinje E S96 (P205), S107
(P242)
British Journal of Cancer (2002) 86(Suppl 1), S122 - S128
 2002 Cancer Research UK All rights reserved 0007 - 0920/02 $25.00
www.bjcancer.com
Chitra A S34 (P3)
Chopada A S97 (P208)
Christian J S13 (1.1)
Chung P S57 (P78)
Chung S S46 (P42)
Clamp A S68 (P113)
Clark AH S43 (P31)
Clark GWB S51 (P59)
Clark S S35 (P6), S78 (P146)
Clarke AR S5 (S8)
Clarke IA S26 (6.4), S107
(P241), S117 (P275)
Clarke NW S64 (P101), S66
(P106), S67 (P109)
Clarke P S49 (P51)
Clarke PA S48 (P50), S89
(P181)
Clarkson L S14 (1.6)
Clawson S S25 (6.1)
Clayton J S26 (6.2)
Clenton SJ S55 (P71)
Clive S S50 (P54)
Clooney K S43 (P31)
Clynes M S62 (P93)
Cody N S91 (P189)
Coffey RNT S66 (P108), S101
(P220)
Coleman JM S65 (P102)
Coleman RE S19 (3.6), S45
(P37), S65 (P102)
Coley HM S22 (4.8)
Collie-Duguid ESR S120
(P285)
Colligan S S57 (P78)
Collingridge DR S13 (1.2)
Collingwood M S25 (5.7)
Colston KW S83 (P161)
Conneally E S88 (P180)
Connolly E S21 (4.5)
Conroy M S72 (P124)
Conte PF S114 (P266)
Cook D S107 (P241)
Cook G S13 (1.1)
Cook S S85 (P168)
Cooke P S74 (P131)
Cookson JC S113 (P263)
Cooper PA S116 (P270)
Coralli C S99 (P214)
Corbo M S16 (2.4), S115
(P269)
Corcoran M S47 (P46)
Cornelius VK S40 (P24)
Cornforth S S100 (P217)
Cornwell H S73 (P128)
Corrie P S47 (P46), S71
(P122), S73 (P128)
Corrihons F S105 (P233)
Cossins J S83 (P162)
Costello SSP S34 (P1)
Cotter FE S11 (S29), S16 (2.4),
S115 (P269)
Cotter T S16 (2.3)
Coulson J S33 (8.8)
Coulson JM S61 (P92)
Court W S96 (P203)
Cowan E S53 (P63)
Cowan R S66 (P106)
Cowel RL S104 (P231)
Cowen ME S59 (P84)
Cowen R S107 (P242)
Cowen RL S29 (7.4), (7.5), S96
(P205), S97 (P206)
Cox G S28 (7.1), S59 (P85),
S97 (P207)
Cox K S34 (P4)
Cox S S60 (P87)
Craig C S94 (P198)
Craig R S90 (P186)
Crampe M S40 (P23)
Crawford M S78 (P144)
Crilly T S72 (P124)
Crnogorac-Jurcevic T S76
(P138)
Croke DT S119 (P280)
Crosbie J S47 (P47), S65
(P104)
Crosby K S77 (P143)
Crow JC S45 (P38)
Crown J S87 (P176)
Cruickshank ME S24 (5.6)
CUETO Group S23 (5.1)
Cullen M S25 (6.1)
Cullinane C S95 (P202)
Cunningham D S1 (CT4), S8
(S18)
Currid CA S74 (P132)
Cutress R S91 (P187)
Cutress RI S32 (8.4)
Czajka A S117 (P275)
Dachs GU S109 (P246)
Dachs GU S99 (P214)
Dalal S S95 (P201)
Dalgleish A S108 (P244)
Dalgleish AG S26 (6.4), S107
(P241), S109 (P247),
(P248), (P250), S117
(P275)
Dallman C S90 (P185)
Daly PA S91 (P189)
Danson S S68 (P113)
d'Ariggo C S35 (P6), (P8)
Darvill N S103 (P229)
Daugaard G S19 (3.5)
Daujat S S8 (S20)
Davies AJ S26 (6.2), S40 (P24)
Davies F S1 (CT1)
Davies SE S101 (P222)
Davies-Humphries J S43 (P33)
Dawson LA S86 (P172)
De Bono JS S50 (P56), S110
(P252), (P253)
Deakin DP S26 (6.2)
Dean RA S17 (2.5), S48 (P50),
S89 (P181)
Dearling JLJ S112 (P257)
Dearnaley D S24 (5.4)
Decatris M S65 (P103)
Decatris MP S69 (P117)
Delaney PV S74 (P133)
Denham J S72 (P125)
Dervan PA S23 (5.3), S34 (P1),
S74 (P132)
Desel C S109 (P250)
Devery R S82 (P157), S84
(P166)
Dexter S S51 (P59)
Di Salvo A S103 (P228)
Dibling B S101 (P221)
Dickson EJ S53 (P63), S94
(P198), S120 (P284)
Dillon JF S47 (P45)
Dixon M S40 (P22)
Dixon MF S48 (P49)
Dixon S S27 (6.8)
Djeha H S30 (7.6)
Dobson PRM S114 (P264)
Doherty A S88 (P178), S105
(P235), S106 (P236)
Doherty AP S30 (7.7)
Dookeran KA S39 (P20), (P21)
Doolan P S62 (P93)
Dopson F S56 (P75)
Double JA S83 (P163), S104
(P230), S112 (P259), S116
(P270)
Douglas DA S80 (P151)
Douglas S S40 (P22)
Douglas-Jones AG S38 (P18)
Douglass HO S1 (CT3)
Dowe A S24 (5.4)
Dowsett M S4 (S5), S37 (P12)
Dredge K S26 (6.4), S117
(P275)
Drengler R S110 (P253)
Druker BJ S7 (S14)
Drummond MW S18 (3.4)
Du Y S13 (1.3)
Dube M S49 (P53)
Duddy PM S70 (P119)
Duffy MJ S36 (P10), S37
(P14), S87 (P175), (P176),
S98 (P213)
Duggan C S87 (P175)
Duncan R S12 (S33)
Dunlop D S50 (P54)
Dunlop DJ S60 (P88)
Dunn JA S1 (CT3)
Durrant LG S38 (P17), S49
(P53)
Earl H S34 (P4)
Earl HM S71 (P121)
Easdale S S49 (P51)
Easton D S70 (P119)
Easton DF S24 (5.4)
Easty DJ S74 (P132)
Eatock M S50 (P54)
Eccles DM S70 (P119)
Eccles SA S96 (P203)
Echeta C S42 (P30)
Edwards J S92 (P190)
Edwards JG S59 (P85), S97
(P207)
Edwards S S24 (5.4)
Eeles R S24 (5.4), S81 (P155)
Eeles RA S70 (P119)
Eisen T S1 (CT2), S18 (3.2)
Eisenman RN S31 (8.1)
El-Emir E S112 (P257)
El-Modir A S66 (P105), S72
(P126)
El-Omar EM S8 (S19)
Elahi MM S42 (P28)
Ellis I S19 (3.6), S30 (7.6),
(7.7), S38 (P17)
Ellis T S75 (P136)
Emberton M S66 (P107)
EORTC GU Group S23 (5.1)
Eskdale J S94 (P198), S120
(P284)
Esmailzadeh H S39 (P21)
Evans GS S94 (P197)
Evans PR S24 (5.4)
Evans TRJ S103 (P227)
Everard J S45 (P37)
Everett SA S107 (P240)
Eves P S94 (P197)
Fairlamb D S66 (P105)
Fairlamb DJ S2 (CT6)
Fan Z S116 (P271)
Farmer PB S112 (P260), S116
(P270)
Farmery S S22 (4.7)
Fenci I S79 (P149)
Feng J S107 (P242)
Fennell DA S16 (2.4), S115
(P269)
Fentiman IS S35 (P6), (P7),
(P8)
Fenton SL S92 (P192)
Fernie NL S53 (P65)
Ferrigno PK S78 (P144), S118
(P277)
Field J S75 (P136)
Field JK S119 (P281)
Fielding J S71 (P123)
Figueroa J S50 (P56)
Finn P S9 (S24)
Finn PW S16 (2.2)
Finnbladder and Norwegian
Bladder Cancer Study
Group S23 (5.1)
Fisher M S19 (3.5)
Fisher PM S55 (P71)
Fitzpatrick J S23 (5.3), S66
(P108)
Fitzpatrick JM S22 (4.6), S64
(P99), S81 (P154), S101
(P220)
Flanagan E S42 (P30)
Fleming FJ S36 (P10)
Foran E S119 (P280)
Forastiere AA S7 (S15)
Forster MD S38 (P15)
Foster B S11 (S30)
Foulis PR S116 (P271)
Fountoulakis A S51 (P58)
Fox EJP S32 (8.6), S74 (P132)
Frame MC S76 (P140), S119
(P279)
Francis RJ S100 (P218), S102
(P223)
Fraser M S26 (6.5)
Fresco L S25 (5.7)
Frisken I S100 (P218)
Frith P S114 (P265)
Fritzer-Szekeres M S115 (P268)
Frush T S39 (P21)
Fuks F S8 (S20)
Fuller C S68 (P112)
Author Index
S123
 2002 Cancer Research UK British Journal of Cancer (2002) 86(Suppl 1), S122 - S128
Fullerton N S59 (P82)
Gabra H S24 (5.5), S90 (P184)
Galetta D S61 (P89)
Gallagher G S94 (P198), S120
(P284)
Gallagher WM S32 (8.6), S74
(P132), S98 (P213)
Gammell SJ S45 (P38)
Ganesan R S74 (P131)
Ganesan TS S27 (6.7), S42
(P30)
Gardner N S116 (P271)
Gaskell M S116 (P270)
Gately K S16 (2.3), S88 (P180)
Gazzard B S19 (3.5), S60
(P87), S63 (P97)
Gebbia V S61 (P89)
Gebril N S114 (P264)
Geh I S64 (P100)
Geh JI S53 (P64)
George D S5 (S6)
George J S65 (P102)
Gescher A S112 (P258)
Gilbert J S30 (7.7)
Gilbertson R S2 (CT7)
Gilchrist R S70 (P119)
Gilmore PG S85 (P169)
Gilmore PM S4 (S4)
Giridharan S S53 (P64), S61
(P90)
Girling DJ S62 (P94)
Glasspool RM S103 (P227)
Gleeson NC S46 (P40)
Glen H S60 (P88)
Glennie MJ S13 (1.3)
Glynne-Jones R S52 (P62), S58
(P79)
Godden J S27 (6.6), S50 (P54)
Goff L S78 (P145)
Goldie V S46 (P42)
Golding BT S111 (P255)
Goldman JP S10 (S27)
Gomez-Roman N S31 (8.1)
Gore M S27 (6.8)
Gorey TF S22 (4.6)
Govinda S S117 (P275)
Gower N S2 (CT6)
Gower NH S1 (CT2)
Grace A S48 (P48)
Graham CL S118 (P276)
Graham S S18 (3.4), S27 (6.6)
Grainger M S19 (3.7)
Grandori C S31 (8.1)
Granesan TS S38 (P15)
Grant K S47 (P46)
Gray L S19 (3.7)
Greco O S99 (P214), S109
(P246)
Green AJ S91 (P189), S100
(P218), S102 (P223), S112
(P257)
Green JA S25 (5.7), S43 (P31),
(P32), (P33)
Green NK S105 (P235)
Greene LM S32 (8.6), S98
(P213)
Greenhalgh R S107 (P241)
Greenman J S59 (P84), S102
(P224)
Greer ML S107 (P240)
Gregor A S10 (S25), S73
(P127)
Gregory W S1 (CT2)
Grieve R S57 (P78)
Griffin RJ S111 (P255)
Grigg A S1 (CT4)
Grigor KM S92 (P190)
Grimer R S95 (P202)
Grover A S20 (3.8)
Guichard Y S33 (8.7), S112
(P260)
Gullick WJ S10 (S27)
Gulmann C S48 (P48)
Guppy AE S44 (P35)
Gupta N S14 (1.5)
Gutcher S S19 (3.6)
Haber DA S4 (S4)
Halanek N S109 (P250)
Halkidou K S85 (P168)
Hall GD S14 (1.6)
Hall M S52 (P62)
Hallissey M S71 (P123)
Hamed H S35 (P6), (P7), (P8)
Hammond LA S50 (P56), S110
(P252), (P253)
Han C S27 (6.7)
Hanby AM S35 (P8)
Hancock B S1 (CT4), S25 (6.1)
Hancock BW S27 (6.8), S41
(P26), S45 (P37), S65
(P102)
Hancock H S41 (P26)
Hao D S110 (P253)
Hardcastle A S79 (P147)
Hardcastle IR S111 (P255)
Hardie LJ S48 (P49), S51 (P59)
Harkin DP S4 (S4), S21 (4.4),
S85 (P169)
Harmer C S13 (1.1)
Harmey J S21 (4.5), S79
(P148), S98 (P212)
Harmey JH S98 (P211)
Harney J S56 (P74), (P75)
Harper PG S1 (CT2)
Harris AL S27 (6.7), S28 (7.2),
S29 (7.4), S38 (P15), S100
(P219), S114 (P265),
(P266)
Harris JC S48 (P50)
Harris M S26 (6.2)
Harris P S30 (7.6), (7.7)
Harris R S47 (P46)
Harris RI S71 (P122)
Harris RM S36 (P11)
Harrison GM S82 (P158)
Harrow S S26 (6.5)
Hart IR S76 (P138), S120
(P282)
Hartley A S53 (P64), S61 (P90)
Hassan S S77 (P142)
Hatton MQ S55 (P71), S65
(P102)
Hawkin R S60 (P87)
Hawkins K S1 (CT1)
Haycock JW S94 (P197)
Hayes NLV S10 (S27)
Hayward CE S41 (P26)
Heald RA S113 (P263)
Hedley S S115 (P267)
Heng YM S94 (P199), S95
(P200)
Hennequin LF S99 (P216)
Henning IM S14 (1.6)
Herbertson R S41 (P27)
Hesson LB S92 (P192)
Hetherington J S102 (P224)
Hew WSR S39 (P19)
Hill A S37 (P14)
Hill ADK S35 (P5), S36 (P10),
S87 (P175), (P176), S97
(P209), S98 (P210)
Hill S S30 (7.6)
Hill SA S14 (1.4)
Hiller L S19 (3.7), S74 (P131)
Hilson AJW S100 (P218), S102
(P223)
Hoang TTV S117 (P274)
Hoare S S18 (3.4), S38 (P15)
Hochtl T S115 (P268)
Hodgetts J S68 (P113)
Hodgson SV S70 (P119)
Hollywood D S16 (2.3)
Holte H S1 (CT4)
Holyoake T S27 (6.6)
Holyoake TL S18 (3.4)
Honess DJ S118 (P278)
Honeychurch J S13 (1.3)
Horne M S30 (7.6), (7.7)
Horsman JM S65 (P102)
Horvath Z S115 (P268)
Hoskin P S1 (CT4), S14 (1.5)
Hoskin PJ S20 (3.8), S28
(7.3)
Howard J S88 (P180)
Howell S S26 (6.2)
Howell WM S24 (5.4)
Howes G S37 (P12)
Howie A S30 (7.7)
Huang J-D S75 (P135)
Hughes MP S22 (4.8)
Hughes PJ S36 (P11)
Hughes R S54 (P67)
Hughes V S50 (P55)
Hui W-S S75 (P135)
Hull D S30 (7.6)
Humphreys S S37 (P12)
Hupp TR S38 (P16), S39
(P19), S47 (P44), (P45),
S76 (P137), S89 (P183),
S113 (P262)
Hurd P S8 (S20)
Huson S S114 (P265)
Hussain SA S64 (P100), S74
(P131)
Hutchinson JA S109 (P247)
Hutchison G S18 (3.3)
Hylands F S118 (P278)
Ibrahim A S70 (P118)
Ijaz T S117 (P274)
Illidge TM S13 (1.3)
Indar AA S49 (P53)
Ingram A S38 (P16), S76
(P137)
Ingram CE S45 (P37)
Inman I S73 (P128)
Innes H S70 (P118)
Ioncaster J S28 (7.2)
Ives N S27 (6.8)
Izbicka E S110 (P252)
Jack A S1 (CT4)
Jackson DP S37 (P13)
Jackson S S112 (P259)
Jacobs I S42 (P29)
Jaffar M S29 (7.5), S100
(P219), S104 (P231)
James HV S65 (P104)
James LE S1 (CT2)
James N S19 (3.7)
James ND S30 (7.7), S64
(P100), S74 (P131), S88
(P178), S105 (P235), S106
(P236)
James NE S30 (7.6)
James RD S18 (3.3)
Jankowski J S48 (P50)
Jeekel J S1 (CT3)
Jeffries K S69 (P117)
Jenkins A S42 (P30)
Jenkins BL S75 (P134)
Jenkins GJS S73 (P129), (P130)
Jenkins TC S106 (P237)
Jennings SA S106 (P237)
Jethwa P S71 (P123)
Jiang WG S82 (P158)
Joel S S78 (P145), (P146), S103
(P229)
Joel SP S17 (2.7), S41 (P25)
John RJ S109 (P247)
John TRL S108 (P243)
Johnson CG S10 (S27)
Johnson M S19 (3.5)
Johnson P S25 (6.1)
Johnson PWM S13 (1.3), S32
(8.4), S90 (P185), (P186)
Johnston D S46 (P42)
Johnston DA S47 (P45)
Johnston DJ S34 (P1)
Johnston P S31 (8.3), S93
(P196)
Johnston PG S4 (S4), S21
(4.4), S85 (P169)
Johnstone J S42 (P28)
Joiner M S31 (8.3)
Jolly J S48 (P49)
Jones GDD S33 (8.7), S112
(P260)
Jones JL S28 (7.1), S59 (P85),
S97 (P207)
Jones PH S83 (P162)
Jones RJ S119 (P279)
Jones WG S3 (CT8), S23 (5.2)
Jorgensen H S27 (6.6)
Kakani R S103 (P227)
Kakkar AK S97 (P208)
Kallioneimi O S6 (S11)
Kan KS S102 (P225)
Kanthou C S99 (P214)
Author Index
S124
British Journal of Cancer (2002) 86(Suppl 1), S122 - S128  2002 Cancer Research UK
Karl D S115 (P268)
Katerinaki E S94 (P197)
Kaur K S38 (P15)
Kay E S48 (P48)
Kay G S103 (P228), S104
(P230)
Keith WN S18 (3.4), S103
(P227), S106 (P239)
Kelly J S3 (CT8), S23 (5.2)
Kelly ZD S32 (8.6), S74 (P132)
Kendrew J S99 (P216), S100
(P217)
Kennedy M S22 (4.6)
Kennedy RD S85 (P169)
Kennedy S S87 (P176)
Kenny LM S44 (P34)
Keogh A S71 (P123)
Kerin MJ S22 (4.6)
Kernohan N S46 (P42)
Kernohan NM S76 (P137)
Kerr DJ S26 (6.3), S30 (7.6)
Kerr K S61 (P91)
Kettlebourough C S78 (P144)
Khan S S33 (8.8)
Khanna S S69 (P117)
King V S72 (P126)
Kini M S45 (P38)
Kirk CJ S36 (P11)
Kirk D S59 (P82)
Kirkbride P S55 (P71)
Kirwan JM S25 (5.7)
Knaak C S32 (8.6)
Knell V S100 (P218), S102
(P223)
Knox R S11 (S32)
Knox RJ S103 (P227)
Kocienski P S105 (P233)
Koller B S4 (S4)
Koncewicz L S73 (P128)
Kothari A S35 (P7), (P8)
Kouzarides S8 (S20)
Kovacev O S72 (P125)
Krishna NS S92 (P190)
Kristaleit H S26 (6.4)
Kriste AG S30 (7.8)
Kumar P S87 (P174)
Kvols L S116 (P271)
Kyrtopoulos SA S106 (P238)
Labeed FH S22 (4.8)
Ladner RD S120 (P285)
Laker R S65 (P104)
Lam T S102 (P224)
Lamb D S72 (P125)
Lane DP S4 (S1)
Langan J S75 (P136)
Langan JE S119 (P281)
Langford L S33 (8.7)
Lankester KJ S14 (1.4), S99
(P215)
Lashford L S2 (CT7)
Last KW S40 (P24)
Latham J S88 (P178)
La Thangue N S9 (S24)
La Thangue NB S16 (2.2)
Latif F S92 (P192)
Latif T S21 (4.4)
Lattmann E S17 (2.6)
Lawler M S16 (2.3), S21 (4.3),
S40 (P23), S88 (P180)
Lawrance JA S26 (6.2)
Lawrie LC S54 (P68)
Leach MO S99 (P215)
Leader M S48 (P48)
Leahy JJ S111 (P255)
Leahy MG S3 (CT8), S23 (5.2)
Lee A S35 (P5), S97 (P209),
S98 (P210)
Lee R S32 (8.4)
Lee SM S1 (CT2)
Legge J S61 (P91)
Lemoine NR S20 (4.2)
Leonard RCF S40 (P22)
Leong C-O S116 (P270)
Le Pla RC S112 (P260)
Leung HCM S82 (P159)
Leung HY S30 (7.7), S31 (8.2),
S75 (P134), S80 (P151)
Le Vay JH S56 (P73)
LeVay J S65 (P104)
Levine E S18 (3.3)
Lewis I S95 (P202)
Li H S87 (P174)
Liang Y S62 (P93)
Liem AA S39 (P19), S89
(P183), S113 (P262)
Lim P S81 (P153)
Linch DC S1 (CT4)
Lind M S59 (P84)
Lister TA S26 (6.2), S40 (P24)
Liu WM S17 (2.7), S41 (P25),
S78 (P145)
Livingstone A S59 (P83)
Lo N S61 (P90)
Lo YMD S6 (S10)
Loadman PM S112 (P259)
Lofts F S49 (P52)
Logan IR S87 (P177)
Logue J S66 (P106)
Lolljee J S59 (P85)
Lomax L S68 (P113)
Long AC S119 (P280)
Longley DB S21 (4.4)
Lorigan P S68 (P113), S115
(P267)
Lorigan PC S94 (P197)
Love C S40 (P22)
Lucraft H S2 (CT7)
Lunec J S91 (P188)
Lunt SJ S96 (P204)
Luo ZJ S75 (P135)
Lynch FJ S120 (P285)
Lynch M S21 (4.4)
Lyons J S66 (P106)
MacDicken IA S43 (P32)
Macdonald AG S67 (P110)
Mackay SP S105 (P234)
Mackean M S70 (P120)
Mackean MJ S53 (P65)
Mackenzie CM S65 (P104)
Mackenzie LM S56 (P73)
Mackintosh DT S27 (6.7)
MacLean AB S45 (P38)
MacLennan K S1 (CT4), S80
(P152)
MacNeil S S94 (P197)
Madden FJ S65 (P103)
Madhava K S61 (P90)
Madhusudan S S38 (P15), S42
(P30), S114 (P265), (P266)
Madjd Z S38 (P17)
Maguire TM S37 (P14)
Maher ER S92 (P192)
Mairs RJ S57 (P77), S59 (P82),
(P83), S89 (P182), S106
(P239)
Maison A S32 (8.4)
Maitland NJ S80 (P150), S86
(P172)
Makrogianelli K S78 (P145)
Malinovszky KM S40 (P22)
Mallucci L S21 (4.3), S85
(P167)
Man H-W S117 (P275)
Mangham C S95 (P202)
Mansel RE S2 (CT5), S15
(1.7), S38 (P18)
Mansi JL S83 (P161)
Mantavani A S112 (P257)
Maraveyas A S49 (P52), S102
(P224)
Marples B S31 (8.3)
Marriott JB S26 (6.4), S117
(P275)
Marshall E S70 (P118)
Marshall JF S120 (P282)
Marson C S78 (P146)
Martin IG S51 (P58)
Martin N S111 (P255)
Martin SG S58 (P80), S108
(P245)
Martin WMC S63 (P98)
Maslove L S2 (CT6)
Mason MD S82 (P158)
Massie C S24 (5.5)
Matthews CS S117 (P273)
Matthews J S26 (6.2)
Mautner V S30 (7.6), (7.7)
Mawdsley S S52 (P62), S58
(P79)
Maxwell PJ S14 (1.4), S21
(4.4), S99 (P215)
Mayer A S117 (P272)
Mays T S110 (P253)
McArdle CS S42 (P28)
McBrierty D S79 (P148)
McCabe N S4 (S4), S85 (P169)
McCann A S23 (5.3)
McCann S S88 (P180)
McCann SR S16 (2.3), S40
(P23)
McCarron SL S24 (5.4)
McCluskey AG S57 (P77)
McConkey C S53 (P64)
McCormack M S66 (P107)
McCusker K S105 (P233)
McDermott E S35 (P5), S37
(P14), S87 (P175), (P176),
S91 (P189), S97 (P209), S98
(P210), (P213)
McDermott EW S36 (P10)
McDermott U S44 (P34)
McDevitt T S91 (P189)
McDonald AC S50 (P55)
McDonnell S S62 (P93), S74
(P133), S82 (P157), (P160)
McEleny KR S66 (P108)
McElwaine S S16 (2.3), S21
(4.3)
McEwan E S47 (P47)
McFadyen M S32 (8.5)
McFadyen MCE S84 (P165),
S111 (P254)
McGoldrick A S23 (5.3)
McGrath D S50 (P54)
McGuinnes EPJ S46 (P40)
McIntosh RA S120 (P285)
McKay JA S120 (P285)
McKay T S20 (4.2)
McKenzie-Edwards E S70
(P119)
McLean C S53 (P66)
McLeish R S43 (P32)
McLeod HL S32 (8.5), S88
(P178)
McMahon G S95 (P201)
McMillan DC S42 (P28)
McMillan TJ S81 (P155)
McNeish IA S20 (4.2)
McRonald F S75 (P136)
McWilliam P S119 (P280)
McWilliams S S4 (S4)
Meade A S77 (P142)
Mehta PB S75 (P134), S80
(P151)
Melillo G S97 (P206)
Melnick M S77 (P143)
Melton R S118 (P276)
Melvin WT S111 (P254)
Meredith D S113 (P261)
Meyer C S34 (P2)
Micallef IN S26 (6.2)
Michael A S49 (P52), S108
(P244)
Michels J S90 (P186)
Middleton MR S68 (P113)
Midgley RS S26 (6.3)
Mikol C S78 (P146)
Millar J S50 (P54)
Miller A S84 (P166)
Miller EP S24 (5.5), S90
(P184)
Miller N S91 (P189)
Mills JA S57 (P78)
Milroy R S2 (CT6)
Mincher DJ S12 (S34), S103
(P228), S104 (P230), S112
(P259)
Mirza D S30 (7.6)
Mirza N S26 (6.3)
Moffat B S5 (S9)
Mohammed M S74 (P131)
Molife R S115 (P267)
Monaghan A S18 (3.4)
Montgomery PQ S63 (P98)
Moore BD S105 (P233)
Moore H S72 (P126)
Moore JV S64 (P101), S67
(P109)
Moore N S114 (P265)
Morgan DAL S58 (P80)
Author Index
S125
 2002 Cancer Research UK British Journal of Cancer (2002) 86(Suppl 1), S122 - S128
Morgan G S1 (CT1)
Morgan MR S120 (P282)
Morrin M S74 (P133)
Morris AJ S94 (P198)
Morrison A S50 (P54)
Morrissey C S64 (P99)
Moss A S70 (P118)
Moss EL S43 (P33)
Mountain A S30 (7.6)
MRC Advanced Bladder
Cancer Group S23 (5.1)
Muers MF S62 (P94)
Mukherjee A S108 (P245)
Mullan PB S4 (S4), S85 (P169)
Mullee MA S32 (8.4)
Muller GW S26 (6.4), S117
(P275)
Mullerat J S101 (P222)
Munro AJ S47 (P45)
Murphy AA S32 (8.6)
Murphy DG S81 (P154)
Murphy J S84 (P166)
Murray A S115 (P267)
Murray D S74 (P133), S82
(P160)
Murray GI S24 (5.6), S32 (8.5),
S54 (P68), S84 (P165), S107
(P240), S111 (P254), S120
(P285)
Murray JC S94 (P199), S95
(P200)
Murray P S74 (P131)
Murray PA S58 (P81)
Muthana M S115 (P267)
Nakielny R S45 (P37)
Napp V S2 (CT6)
Nash GB S86 (P173)
Nash GF S97 (P208)
NCIC Clinical Trials Group
S23 (5.1)
Neal DE S3 (CT8), S23 (5.2),
S30 (7.7), S31 (8.2), S75
(P134), S80 (P151), S85
(P168), S87 (P177)
Needham G S72 (P124)
Nelson M S19 (3.5), S60 (P87),
S63 (P97)
Nelstrop A S44 (P35)
Neoadjuvant Chemotherapy
for Cervix Cancer
Meta-analysis Collaboration
(NACCMA Collaboration)
S25 (5.8), S44 (P36)
Neoptolemos JP S1 (CT3)
Newbatt Y S79 (P147)
Newbold RF S91 (P188)
Newell DR S118 (P276)
Newton C S102 (P224)
Ng SY S31 (8.2)
Nicolaou A S83 (P163), S106
(P238)
Nicolson M S61 (P91)
Nordic Lymphoma Group S1
(CT4)
Norfolk and Waveney Head
and Neck Cancer Group
S63 (P98)
Norton A S18 (3.2), S26 (6.2),
S40 (P24)
Nussey F S70 (P120)
O'Brien MER S18 (3.2)
O'Brien NA S37 (P14)
O'Byrne K S69 (P117)
O'Byrne KJ S28 (7.1), S59
(P85), S97 (P207)
Ocejo-Garcia M S61 (P92)
Ochoa L S50 (P56)
Ochs J S50 (P56)
O'Connell M S62 (P95)
O'Connell MEA S63 (P96)
O'Connor A S82 (P157)
O'Connor L S16 (2.3)
O'Connor TR S33 (8.7)
O'Donovan N S37 (P14), S87
(P176)
O'Dwyer ST S18 (3.3)
O'Flynn KJ S67 (P109)
Ogilvie DJ S99 (P216)
Oglesby SD S47 (P44), (P45)
O'Hagan J S43 (P31)
O'Hanlon D S22 (4.6)
O'hIci B S91 (P189)
O'Higgins N S35 (P5), S36
(P10), S37 (P14), S87
(P175), (P176), S97 (P209),
S98 (P210), (P213)
Oikonomou E S17 (2.6)
Okines AFC S41 (P26)
Oliver T S19 (3.5), S103
(P229)
Olivo N S50 (P56)
Olliff S S30 (7.6)
Olliver JR S51 (P59)
O'Marcaigh A S40 (P23)
O'Meara A S40 (P23)
O'Neil MA S113 (P262)
O'Neill JW S90 (P186)
O'Neill MA S39 (P19), S89
(P183)
Orakzai RH S46 (P41)
Orakzai SH S46 (P41)
O'Rourke N S60 (P88)
Orridge C S58 (P80)
O'Shea C S87 (P175), (P176)
O'Shea DG S34 (P1)
Osman S S14 (1.5)
O'Toole SA S46 (P40)
Ottely CJ S108 (P243), S111
(P256)
Owens S S26 (6.2)
Pace A S4 (S4)
Packham G S32 (8.4), S90
(P185), (P186), S91 (P187)
Padhani AR S99 (P215)
Pallaska A S16 (2.4), S115
(P269)
Palmer D S26 (6.3)
Palmer DH S30 (7.6)
Pandha H S26 (6.4), S107
(P241), S108 (P244), S109
(P248)
Pandha HS S109 (P247)
Pandolfi PP S9 (S21)
Papanastassiou V S26 (6.5)
Pardo OE S120 (P283)
Parker CA S100 (P219)
Parker CJ S87 (P174)
Parkes A S22 (4.7)
Parkin DE S24 (5.6)
Parkin RL S59 (P83)
Parkins CS S86 (P173)
Parry JM S73 (P129), (P130)
Parsons SL S17 (2.5)
Parton M S37 (P12)
Patel K S80 (P152)
Paterson SC S36 (P9)
Patnaik A S50 (P56), S110
(P252)
Patterson AV S29 (7.5), S104
(P231)
Patterson LH S107 (P240)
Pavillard V S83 (P163)
Payne HA S66 (P107)
Peacock JH S81 (P155)
Peake D S61 (P90)
Peake MD S2 (CT6)
Pearson ADJ S91 (P188)
Pearson DG S108 (P243), S111
(P256)
Pedley RB S112 (P257)
Perkins S S112 (P258)
Perren T S42 (P30), S69
(P115)
Perren TJ S14 (1.6), S37 (P13)
Perrett CW S45 (P38), S101
(P222)
Perry J S78 (P146)
Petrie IA S112 (P257)
Pettersson S S104 (P230)
Petty RD S52 (P61)
Philips H S50 (P54)
Phillips HA S53 (P66)
Phillips RM S83 (P163), S106
(P237)
Phillips VA S106 (P237)
Philpott A S83 (P162)
Pickering LM S83 (P161)
Picton S S68 (P114)
Pinedo HM S114 (P266)
Pletsa V S106 (P238)
Pletsas D S106 (P238)
Plumb JA S16 (2.2), S103
(P227)
Podd TJ S47 (P47)
Polychronis A S26 (6.4)
Ponchel F S22 (4.7)
Ponder S S38 (P17)
Porteous DJ S24 (5.5), S90
(P184)
Porterfield E S73 (P127)
Potter GA S12 (S35), S117
(P274)
Potter MA S53 (P66)
Powles J S63 (P97)
Powles T S19 (3.5), S60 (P87),
S63 (P97)
Poynter AJ S47 (P47), S56
(P73)
Poynter AJ S65 (P104)
Pratt N S46 (P42)
Premachandra DJ S63 (P98)
Prescott S S3 (CT8), S23 (5.2)
Price A S50 (P54)
Price P S110 (P251), S114
(P265)
Price PM S13 (1.2), S14 (1.5)
Priest K S18 (3.2)
Propper DJ S17 (2.7)
Protheroe A S68 (P114), S69
(P115)
Purdy D S117 (P272)
Purohit OP S19 (3.6)
Purushotham AD S34 (P4)
Qian W S62 (P94)
Quigg M S59 (P83)
Quinn F S40 (P23)
Quinn JE S4 (S4), S85 (P169)
Quirke P S10 (S26), S77
(P141), (P142)
Rabiasz GJ S24 (5.5), S90
(P184)
Radford J S25 (6.1)
Radford JA S26 (6.2)
Radstone CR S41 (P26), S65
(P102)
Rainey D S113 (P261)
Raleigh JA S28 (7.2), S112
(P257)
Rampling R S26 (6.5)
Ramus J S34 (P2)
Rastinejad F S11 (S30)
Ratcliffe PJ S29 (7.4)
Raynaud FI S111 (P255)
Rea D S36 (P11), S61 (P90)
Rehemtulla A S5 (S9)
Reid WMN S45 (P38)
Renehan AG S18 (3.3)
Rennie I S115 (P267)
Rice K S95 (P200)
Richardson C S28 (7.1)
Richardson D S28 (7.1), S59
(P85)
Richman SD S77 (P141),
(P142)
Richmond L S27 (6.6)
Rickards D S66 (P107)
Rigoreau L S111 (P255)
Riley P S64 (P100)
Rimmer YL S55 (P70)
Rioja A S78 (P146)
Risk J S75 (P136)
Risk JM S119 (P281)
Robbins RA S95 (P200)
Roberts JT S23 (5.1)
Roberts L S71 (P121)
Roberts SA S81 (P156)
Roberts T S3 (CT8), S23
(5.2)
Robins RA S49 (P53)
Robinson K S2 (CT7)
Robson CN S31 (8.2), S75
(P134), S85 (P168), S87
(P177)
Roche-Nagle G S21 (4.5), S98
(P212)
Rogers SJ S54 (P67)
Rogowski W S39 (P20), (P21)
Author Index
S126
British Journal of Cancer (2002) 86(Suppl 1), S122 - S128  2002 Cancer Research UK
Rohatiner AZR S26 (6.2), S40
(P24), S78 (P145)
Romero MR S16 (2.2)
Rooney PH S24 (5.6), S32
(8.5), S84 (P165)
Rosa HS S8 (S20)
Ross BD S5 (S9)
Ross PJ S6 (S13), S18 (3.2)
Ross SC S89 (P182), S106
(P239)
Ross VG S120 (P285)
Routledge MN S33 (8.7)
Rowbottom L S75 (P136)
Rowinsky EK S50 (P56), S110
(P252), (P253)
Rowlands BJ S17 (2.5)
Royle GT S32 (8.4)
Rudd RM S2 (CT6), S1
(CT2)
Ruparelia KC S117 (P274)
Russell A S120 (P282)
Russell N S109 (P250)
Russell SG S54 (P67)
Russell ST S86 (P171)
Rustin GJS S44 (P35), S99
(P215)
Ryan AJ S99 (P216)
Ryan B S87 (P176)
Ryan J S88 (P180)
Ryder D S25 (6.1)
Ryder K S18 (3.1), S35 (P8)
Salam A S46 (P41)
Saleem A S14 (1.5)
Salem AA S38 (P18)
Sales M S46 (P42)
Samuel L S52 (P61)
Sander R S81 (P153)
Sanderson A S14 (1.6)
Sangar VK S66 (P106)
Santhanam S S57 (P76),
S65 (P103), S69
(P117)
Sapountzi V S87 (P177)
Sarker D S60 (P87)
Sattar N S42 (P28)
Saunders M S56 (P74)
Saunders MI S56 (P75)
Sausville E S97 (P206)
Savarirayan R S75 (P135)
Saxty B S78 (P144)
Sayer V S103 (P229)
Scatchard K S78 (P145)
Schatzlein AG S104 (P232)
Schafer P S117 (P275)
Schatzlein AG S105 (P233),
(P234)
Schneider R S8 (S20)
Scholefield JH S49 (P53)
Schwartz G S50 (P56), S110
(P252)
Schwartzberg L S50 (P56)
Scigalla P S114 (P265)
Sciupokiene E S39 (P20),
(P21)
Scott D S24 (5.5)
Scott GB S33 (8.7)
Scott KA S17 (2.7)
Scottish Audit of Gastric and
Oesophageal Cancer S51
(P57)
Scotto KW S9 (S22)
Scrase CD S65 (P104)
Searle P S30 (7.6)
Searle PF S30 (7.7), S88
(P178), S105 (P235), S106
(P236)
Sebelova S S4 (S4)
Seckl MJ S44 (P35), S120
(P283)
Sekosan M S39 (P20), (P21)
Selby P S1 (CT1), S3 (CT8),
S23 (5.2), S69 (P115), S80
(P152)
Sellar GC S24 (5.5), S90
(P184)
Selvadurai ED S63 (P96)
Senaratne SG S83 (P161)
Seymour MT S77 (P141),
(P142)
Shah P S60 (P87)
Shamash J S103 (P229)
Sharieff S S46 (P41)
Sharma SP S107 (P242)
Sharrard RM S80 (P150)
Shaw J S47 (P46), S73 (P128)
Shaw P S60 (P86)
Shawki H S43 (P31)
Sheppard BL S46 (P40)
Shin F S39 (P21)
Shorrocks J S81 (P155)
Short S S56 (P74), S58 (P79)
Short SC S56 (P75)
Shrotria S S34 (P2), (P3)
Shum DKY S82 (P159)
Sibtain A S28 (7.3)
Siddiqui GK S45 (P38)
Siller C S37 (P13), S68 (P114),
S69 (P115)
Simcock R S62 (P95), S69
(P116)
Simpson A S95 (P202)
Singer J S71 (P121)
Singh N S42 (P29)
Skillman J S35 (P6)
Smith A S69 (P115)
Smith AM S102 (P225)
Smith BL S77 (P143)
Smith GCM S111 (P255)
Smith HJ S86 (P170)
Smith I S37 (P12)
Smith IE S18 (3.2)
Smith J S23 (5.3)
Smith KD S36 (P9)
Smith L S47 (P47), S50 (P56),
S110 (P252), (P253)
Smith NF S111 (P255)
Smith OP S40 (P23)
Smith P S1 (CT4), S25 (6.1)
Smyth J S45 (P39)
Smyth JF S24 (5.5), S90 (P184)
Soomro I S61 (P92)
Southern S S43 (P32)
Southgate C S24 (5.4)
Speight PM S120 (P282)
Speirs V S22 (4.7)
Spellacy N S21 (4.3)
Spencer DIR S117 (P272)
Spencer JA S14 (1.6)
Spiro S S2 (CT6)
Spiro SG S1 (CT2)
Spittle MF S54 (P67)
Sratford IJ S104 (P231)
Srihari N S53 (P64)
Sriwardhana C S37 (P13)
Stallings R S40 (P23), S88
(P180)
Stanley LA S117 (P274)
Stanton C S82 (P157), S84
(P166)
Stat C S35 (P8)
Stebbing J S19 (3.5)
Steele PRM S43 (P33)
Stenning S S25 (6.1)
Stephens R S77 (P142)
Stephens RJ S2 (CT6)
Stevens A S57 (P78)
Stevens M S4 (S3)
Stevens MFG S108 (P245),
S113 (P263), S116 (P270),
S117 (P273)
Stevenson GT S102 (P225)
Steward WP S69 (P117), S112
(P258)
Stewart LA S25 (5.8), S44 (P36)
Stezhka L S71 (P123)
Stimson L S79 (P147)
Stirling A S19 (3.7)
Stirling D S26 (6.4), S117
(P275)
Stirling JJ S99 (P215)
Stocken DD S1 (CT3)
Stockley ML S111 (P255)
Stocks SC S46 (P42)
Stoll V S20 (4.2)
Stratford I S96 (P205), S107
(P242)
Stratford IJ S28 (7.2), S29
(7.4), (7.5), S96 (P204), S97
(P206), S100 (P219)
Stratford MRL S109 (P246)
Strathdee G S20 (4.1)
Strauss SJ S40 (P24)
Stribbling SM S30 (7.8)
Stronach EA S24 (5.5), S90
(P184)
Stuart D S103 (P226)
Stuart NSA S68 (P112)
Stuart R S46 (P42), S51 (P57)
Stuart RC S53 (P63), S94
(P198), S120 (P284)
Sue-Ling HM S51 (P58)
Swaine DJ S83 (P163)
Swinson D S97 (P207)
Swinson DEB S28 (7.1), S59
(P85)
Sydes M S25 (6.1)
Symonds P S25 (5.7), S94
(P199), S95 (P200)
Symonds RP S69 (P117)
Szekeres T S115 (P268)
Talbot D S38 (P15)
Talbot DC S27 (6.7)
Tan HL S117 (P274)
Taylor CT S101 (P220)
Taylor GA S118 (P276)
Taylor HJH S55 (P72)
Taylor NJ S99 (P215)
Taylor R S2 (CT7)
Teh SH S35 (P5), S97 (P209),
S98 (P210)
Telfer BA S29 (7.5), S104
(P231)
Tenev T S20 (4.2)
Tercel M S30 (7.8)
Thatcher N S68 (P113)
The CRC/BPG UK Familial
Prostate Cancer Study
Collaborators S24 (5.4)
Thomas A S69 (P117)
Thomas G S57 (P76)
Thomas GA S76 (P139)
Thomas GJ S120 (P282)
Thomas HD S118 (P276)
Thompson A S7 (S17)
Thompson AM S38 (P16), S39
(P19), S46 (P42), S46
(P43), S47 (P44), (P45),
S51 (P57), S72 (P124), S76
(P137), S89 (P183), S113
(P262)
Thomson C S41 (P26)
Thornton H S34 (P4)
Thorpe H S2 (CT6)
Thorpe N S57 (P78)
Tierney JF S25 (5.7), (5.8)
Tierney JF S44 (P36)
Tilby MJ S84 (P164), S86
(P170), (P171), S91 (P188),
S108 (P243), S111 (P256)
Titley J S49 (P51)
Tobi SE S81 (P155)
Todd I S95 (P200)
Tolcher AW S110 (P252),
(P253)
Tolner B S117 (P272)
Townsend P S91 (P187)
Townsend PA S32 (8.4)
Tozer GM S14 (1.4), S86
(P173), S99 (P214), S109
(P246), S118 (P278)
Tran H S39 (P20), (P21)
Trapani V S116 (P270)
Trott DA S91 (P188)
Tsolomas M S114 (P265)
Tupper J S109 (P246)
Turnbull A S104 (P230), S112
(P259)
Turner AJ S86 (P172)
Turner L S19 (3.6)
Turner LA S27 (6.8)
Turner M S109 (P248)
Twal WO S98 (P213)
Twelves C S103 (P226)
Ubhi T S113 (P261)
Uchegbu IF S104 (P232)
Uchegbu IF S105 (P234)
UK Lymphoma Group S1 (CT4)
UKLG LY09 Collaborators S25
(6.1)
Author Index
S127
 2002 Cancer Research UK British Journal of Cancer (2002) 86(Suppl 1), S122 - S128
Underwood MA S92 (P190)
Usmani BA S86 (P172)
Valenza R S61 (P89)
Vasanthan S S57 (P76), S69
(P117)
Vasselin DA S117 (P273)
Vernon AE S83 (P162)
Vini L S55 (P72)
Vinnicombe S S26 (6.2)
Vojnovic B S81 (P156)
Waite KJ S54 (P69)
Wall L S45 (P39)
Wallace DMA S30 (7.7), S88
(P178), S105 (P235), S106
(P236)
Waller D S2 (CT6)
Wang W S88 (P178)
Wanogho E S117 (P274)
Warbrick E S47 (P45)
Ward SJ S109 (P250)
Ward W S95 (P200)
Wardman P S81 (P156), S109
(P246)
Waring RH S36 (P11)
Waters JS S18 (3.2)
Watkins CJ S16 (2.2)
Watson RWG S22 (4.6),
S64 (P99), S66 (P108),
S81 (P154), S101
(P220)
Watson SA S17 (2.5), S48
(P50), S89 (P181)
Watt KP S24 (5.5)
Waugh D S93 (P196)
Wedge SR S99 (P216), S100
(P217)
Wells G S117 (P273)
Wells V S21 (4.3), S85 (P167)
WelshPS89(P182),S106(P239)
West CML S28 (7.2)
Westhoff MA S76 (P140)
Weston C S2 (CT7)
Westwell AD S108 (P245),
S117 (P273)
Wheatley K S27 (6.8)
Wheelhouse RT S106 (P237),
(P238)
Whelan M S109 (P250)
Whitaker R S68 (P112)
White J S60 (P88)
White KLM S51 (P58)
White RJ S31 (8.1)
Whitehouse AS S84 (P164)
Wild CP S48 (P49), S51 (P58),
(P59)
Wilkinson D S115 (P267)
Williams CJ S25 (5.7)
Williams GL S73 (P129),
(P130)
Williams KJ S29 (7.4), (7.5),
S96 (P204), S97 (P206),
S100 (P219), S104 (P231)
Williams R S9 (S24), S68
(P112)
Williams RJ S16 (2.2)
Williams RN S17 (2.5)
Williamson K S66 (P108)
Wilner S S42 (P30)
Wilsher NE S117 (P274)
Wilson A S40 (P24)
Wilson CB S47 (P46)
Wilson D S19 (3.7), S57 (P78)
Wilson E S73 (P127)
Wilson GD S28 (7.3), S81
(P153), S107 (P240)
Wilson H S13 (1.2)
Wilson LE S59 (P83)
Wilson WR S30 (7.8)
Wind N S107 (P242)
Winslet MC S101 (P222)
Woenckhaus J S79 (P149)
Wolf CR S39 (P19), S89
(P183), S113 (P262)
Woll PJ S33 (8.8), S61 (P92)
Wong Te Fong LF S45 (P38),
S101 (P222)
Wood DM S36 (P11)
Wood L S32 (8.4)
Woodcock M S31 (8.3)
Woodman R S118 (P277)
Wooster R S49 (P51)
Workman P S49 (P51), S79
(P147), S96 (P203), S111
(P255)
Wrighton C S30 (7.6)
Wyke AW S119 (P279)
Wylie J S66 (P106)
Yang J S39 (P20), (P21)
Yang L S75 (P135)
Yarnold J S5 (S7)
Yau K S110 (P251)
Yoneda M S46 (P40)
Young AE S18 (3.1)
Young B S78 (P146)
Young I S71 (P123)
Young JG S30 (7.7), S88
(P178), S105 (P235), S106
(P236)
Young L S26 (6.3), S35 (P5),
S36 (P10), S97 (P209), S104
(P230)
Young LS S30 (7.6), S88
(P178), S105 (P235), S106
(P236)
Yung L S1 (CT4)
Zalutsky MJ S89 (P182), S106
(P239)
Zaren HA S39 (P20), (P21)
Zhang Y S83 (P162)
Zhao J S103 (P227)
Zhu G S76 (P138)
Zhu N S94 (P197)
Zinselmeyer BH S105 (P234)
Ziyaie D S38 (P16), S76 (P137)
Author Index
S128
British Journal of Cancer (2002) 86(Suppl 1), S122 - S128  2002 Cancer Research UK

				

